# Acceptabilité du vaccin antivirus du papillome humain: enquête auprès des parents

**DOI:** 10.11604/pamj.2018.31.71.15400

**Published:** 2018-10-02

**Authors:** Naima Baddouh, Noureddine Rada, Fatima Ezzahra Elalouani, Ghizlane Draiss, Mohammed Bouskraoui

**Affiliations:** 1 Service de Pédiatrie A, CHU Mohammed VI, Marrakech, Faculté de Médecine et de Pharmacie, Université Cadi Ayyad, Marrakech, Maroc

**Keywords:** Acceptabilité, cancer du col de l´utérus, enquête, vaccin anti HVP, virus du papillome humain, Acceptability, cancer of the cervix, survey, HVP vaccine, human papillomavirus

## Abstract

Le but de ce travail est d'évaluer les connaissances des parents des filles en âge de vaccination sur le vaccin anti virus du papillome humain (HPV), leur acceptabilité du vaccin et les facteurs associés au refus. Nous avons mené une enquête auprès des parents de filles âgées de 8 à 15 ans, suivies pour diverses pathologies au service de pédiatrie du CHU Mohamed VI de Marrakech, Maroc, portant sur le profil des parents, leur connaissances sur le cancer du col de l'utérus, l'HPV, et le vaccin anti HPV, l'acceptabilité de vacciner leur filles et les arguments liés au refus. Quatre vingt seize questionnaires ont été inclus dans l'analyse. Le cancer du col est considéré fréquent pour 58% des parents. Seuls 5% connaissaient le vaccin anti HPV. Leur source d'information à tous était les médias. Personne n'avait d'idée sur le coût du vaccin et sa tolérance. Aucune fille n'était vaccinée contre l'HPV. Soixante trois pour cent des parents voudraient bien vacciner leurs filles, ce taux a augmenté à 82% après sensibilisation des parents. Treize pour cent des parents étaient hésitants alors que 24% ont refusé de vacciner pour cause d'effets secondaires majoritairement (51%). Les parents refusant le vaccin étaient en majorité de sexe masculin, de niveau socio-économique et culturel moyen, et ignoraient le virus et le vaccin dans 91% des cas. Cette étude a permis de soulever les éléments de réticences face au vaccin anti HPV afin d'optimiser les stratégies de communication auprès des parents.

## Abstract (en)

This study aims to evaluate the extent of human papilloma virus vaccine awareness among parents of girls in vaccine age group, their acceptability of the vaccine and factors associated with refusal. We conducted a survey among parents of girls aged 8-15 years, followed-up for several diseases in the Department of Pediatrics at the University Hospital Mohamed VI in Marrakech, Morocco, on parents' profile, their awareness of cancer of the cervix, HPV and HPV vaccine, the acceptance of HPV vaccine for their daughters and the arguments related to refusal. Ninety six questionnaires were included in the analysis. Cancer of the cervix was considered frequent for 58% of parents. Only 5% of parents knew about HPV vaccine. Media were the source of information in all cases. Nobody had no idea about the cost of the vaccine and its tolerance. No girl was vaccinated against HPV. Sixty-three per cent of parents want their daughters to be vaccinated, this rate increased by 82% after awareness. Thirteen per cent of the parents were hesitant while 24% refused to vaccinate their daughters mainly due to side effects (51%). Parents refusing vaccine were predominantly males with medium socioeconomic status and cultural level and were unaware of the virus and the vaccine in 91% of cases. This study highlights the reasons for parents' reluctance towards HPV vaccine in order to optimize strategies for effective communication with parents.

## Aux éditeurs du Journal Panafricain de Médecine

Le cancer du col de l'utérus représente la deuxième cause de décès par cancer chez la femme après le cancer du sein. Il est responsable d'environ 270000 décès par an dans le monde [1]. La majorité des cancers du col de l'utérus surviennent chez les femmes des pays en développement du fait de l'absence de dépistage [2, 3]. Au Maroc aussi, le cancer du col représente le deuxième cancer de la femme après celui du sein [4]. Le lien entre certains sérotypes d'HPV et le cancer du col et les condylomes a été clairement établi [1]. Deux vaccins anti HPV sont disponibles pour la prévention de l'infection associée avec les sérotypes 16, 18, 6 et 11 chez les femmes [5]. Malgré l'impact fortement positif de ces deux vaccins, une réticence grandissante leur est associée. Nous avons menu cet enquête pour évaluer l'état des connaissances des parents des filles en âge de vaccination vis à vis du vaccin anti HPV, évaluer leur acceptabilité du vaccin et ressortir les facteurs associés à un avis favorable ou défavorable à la vaccination anti-HPV. C'est une enquête « Connaissances, Attitudes, Pratiques » (dite CAP) réalisée auprès des parents de filles âgées de 8 à 15 ans, suivies à la consultation du service de pédiatrie CHU Mohammed VI Marrakech pour diverses pathologies, entre Septembre 2014 et Janvier 2015. L'échantillonnage a été réalisé sur un mode aléatoire et stratifié en fonction de l'âge. Un questionnaire standardisé anonyme a été administré par un médecin aux parents de l'enfant. Nous avons inclus dans l'analyse 96 questionnaires qui étaient complètement remplis. L'analyse a été faite à l'aide des statistiques descriptives sur EXCEL.

**Figure 1:**
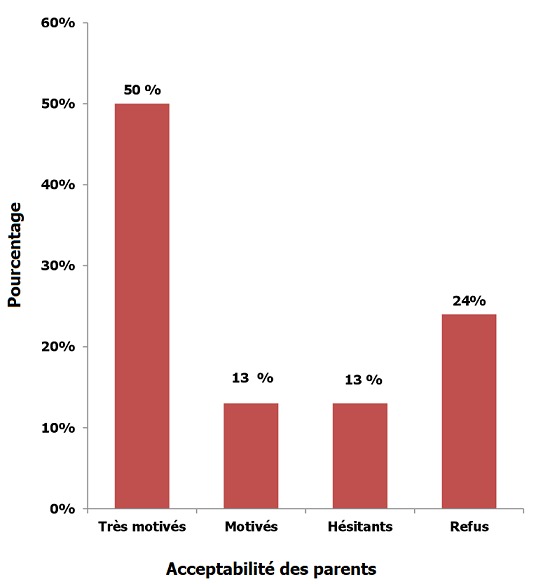
Acceptabilité des parents par rapport au vaccin

L'âge des filles variait entre 8 et 15 ans avec une moyenne d'âge de 10 ans. Elles étaient accompagnées par la mère dans 78% des cas, et étaient toutes vaccinées selon le programme national d'immunisation en secteur public. Les caractéristiques sociodémographiques des participantes sont résumées dans le Tableau 1. Le cancer du col est considéré pathologie fréquente dans notre contexte, seulement, pour 58% des parents. Seuls 5% des parents connaissaient le virus, le définissant comme facteur de risque du cancer du col et savaient qu'il est sexuellement transmissible; Tous ayant eu des études supérieures. Personne ne savait que l'homme peut être un porteur sain. De même 5% des parents connaissaient le vaccin anti HPV, et savaient qu'il est disponible au Maroc (en pharmacie). Leur source d'information à tous était les médias. Personne n'avait d'idée sur le coût du vaccin, ni sur sa tolérance. Aucune fillette n'était vaccinée contre le HPV. Soixante trois pour cent des parents voudraient bien vacciner leurs filles dont 50% étaient très motivés (Figure 1). Ce taux augmente à 82 % après avoir eu des informations sur le lien entre le cancer du col et l'HPV et sur le vaccin anti HPV. 71% des parents pensaient que le vaccin anti HPV devrait faire partie du calendrier vaccinal marocain. Vingt quatre pour cent des parents refusaient de vacciner leur filles, 9 % parmi eux avaient des doutes sur l'efficacité du vaccin alors que 51% le refusaient par crainte d'effets secondaires et 40 % par manque de moyen (prix du vaccin). Les parents refusant la vaccination contre l'HPV étaient majoritairement de sexe masculin. Tous d'origine géographique urbaine et tous de moyen niveau socio-économique. Tandis que 91% d'entre eux ne connaissaient ni le virus ni le vaccin.

**Tableau 1:** Caractéristiques sociodémographiques des participantes

Variable	Nombre	Pourcentage (%)
**Age**		
8 à 11 ans	41	43
11 à 13 ans	30	31
13 à 15 ans	25	26
**Accompagnant**		
La mère	75	78
Le père	21	22
**Niveau socio-économique des parents**		
Bas	62	65
Moyen	34	35
**Niveau culturel des parents**		
Non scolarisés	12	13
Scolarisés	78	81
Etudes supérieures	6	6
**Origine géographique des parents**		
Urbaine	82	85
rurale	14	15

Dans notre échantillon aucune des filles n'avait reçu le vaccin anti-HPV, alors que le taux de couverture vaccinale dans les pays développés est élevé [1, 6]. Seuls 5% des parents connaissent le virus et savent que le vaccin est dirigé contre ce virus. Il a été prouvé que l'information des parents sur le cancer du col et le HPV accroit considérablement l'acceptabilité du vaccin [2, 6]. Les medias étaient la source d'information pour tous les parents dans notre population, alors que des taux plus élevés d'adhésion au vaccin ont été constatés dans la littérature chez les parents informés par leurs médecins [6]. Les deux tiers des parents acceptent de vacciner leurs filles. Parallèlement, des études faites au Maroc, en Afrique et dans différents pays ont rapportées une grande acceptabilité du vaccin [2, 3, 7, 8]. Après avoir eu des connaissances sur le cancer du col et le HPV, le taux d'acceptabilité du vaccin a augmenté dans notre étude de 63 % à 82%. Même constatation dans d'autres études africaines [2, 9]. Ce qui prouve qu'accroître la confiance des gens sur les vaccins peut influencer pour une meilleure prédisposition à être vacciné. La crainte des effets secondaires reste le premier souci des parents dans notre population, chose qui a été notée aussi dans d'autres études [3, 9, 10]. Les parents de niveau socioéconomique et culturel élevé sont plus réceptifs à avoir le vaccin dans notre étude. Le rôle des facteurs socio-économiques et culturels dans l'acceptabilité du vaccin a été noté aussi dans plusieurs études [2, 3, 7, 8]. Le refus de la vaccination dans notre enquête est corrélé à une origine urbaine des filles, un niveau socio-économique et d'instructions moyennes, la méconnaissance du virus et de la disponibilité du vaccin. Des campagnes de sensibilisation s'avèrent nécessaires pour informer les parents sur la disponibilité du vaccin et ses bénéfices.

## Conclusion

Cette étude a permis de relever le manque d'information des parents concernant la prévention d'une des pathologies les plus fréquentes dans notre contexte. Elle a aussi permis d'identifier les éléments de réticences face à la vaccination anti-Papillomavirus afin d'optimiser les stratégies de communication auprès des parents. L'acceptabilité du vaccin anti HPV semble dépendre de l'information des parents et de la baisse du prix du vaccin.

## Conflits d’intérêts

Les auteurs déclarent ne pas avoir de conflits d'intérêts.
